# *Homo temporus*: Seasonal Cycles as a Fundamental Source of Variation in Human Psychology

**DOI:** 10.1177/17456916231178695

**Published:** 2023-07-10

**Authors:** Ian Hohm, Alexandra S. Wormley, Mark Schaller, Michael E. W. Varnum

**Affiliations:** 1 Department of Psychology, University of British Columbia; 2 Department of Psychology, Arizona State University

**Keywords:** seasons, ecology, mating

## Abstract

Many animal species exhibit seasonal changes in their physiology and behavior. Yet despite ample evidence that humans are also responsive to seasons, the impact of seasonal changes on human psychology is underappreciated relative to other sources of variation (e.g., personality, culture, development). This is unfortunate because seasonal variation has potentially profound conceptual, empirical, methodological, and practical implications. Here, we encourage a more systematic and comprehensive collective effort to document and understand the many ways in which seasons influence human psychology. We provide an illustrative summary of empirical evidence showing that seasons impact a wide range of affective, cognitive, and behavioral phenomena. We then articulate a conceptual framework that outlines a set of causal mechanisms through which seasons can influence human psychology—mechanisms that reflect seasonal changes not only in meteorological variables but also in ecological and sociocultural variables. This framework may be useful for integrating many different seasonal effects that have already been empirically documented and for generating new hypotheses about additional seasonal effects that have not yet received empirical attention. The article closes with a section that provides practical suggestions to facilitate greater appreciation for, and systematic study of, seasons as a fundamental source of variation in human psychology.

In autumn, sockeye salmon embark on an epic journey, swimming up to 600 miles from the ocean to the rivers in which they were born to spawn and die (Crossin et al., 2004). At the same time, Canada geese fly thousands of miles south seeking warmer weather, and then in the spring they return north to breed (Wege & Raveling, 1983). In winter, the black bear enters a state of hibernation, slowing its heart rate to less than 10 beats per minute and remains in hibernation until the spring (Hellgren, 1998). Further north, the arctic fox molts, trading its dark coat of summer fur for a new white one that blends in with the snow and ice (Mills et al., 2018). Every December, children in much of the world make a special effort to be on their best behavior in advance of the arrival of a large, mythical, bearded man in a red suit who delivers gifts in the middle of the night. At first glance, this last seasonal pattern appears to have little in common with our first several examples. Yet the last example illustrates a similar point as those before it—that human behavior, like that of many other organisms, is profoundly influenced by seasons. Indeed, as we discuss below, just as a behavioral description of *Oncorhynchus nerka* (the sockeye salmon) would be incomplete without carefully documenting its seasonal migratory and mating patterns, so too the scientific study of *Homo sapiens* would be incomplete without systematic inquiry into seasonal variation in human psychology.

Some effects of seasons on psychological phenomena are well known, of course—such as seasonal affective disorder (SAD), the clinical condition characterized by recurring depression in winter months (American Psychiatric Association, 2013; Han et al., 2000; Magnusson & Boivin, 2003). But, as we describe below, SAD is just the tip of the proverbial iceberg. Seasons also affect many other psychological phenomena, including decision-making in diverse behavioral domains such as aggression, prosocial behavior, eating behavior, and sexual activity (Kenrick & MacFarlane, 1986; Lauritsen & White, 2014; Levin et al., 2002; Ma et al., 2006; Markey & Markey, 2013; Sachs & Chu, 2000), aesthetic preferences for music and for colors (Pettijohn, Williams, & Carter, 2010; Palmer & Schloss, 2010; Park et al., 2019), as well as attentional processes and executive functioning (Meyer et al., 2016). But, despite these many findings, seasons remain an underappreciated source of variation in psychological phenomena—especially compared with other systematic sources of variation. Entire subfields of psychology have coalesced around other sources of variation, such as variation resulting from individual differences (personality psychology), developmental processes (developmental psychology), situational context (social psychology), and cultural background (cultural psychology). In contrast—despite the potentially profound conceptual, empirical, methodological, and practical implications associated with seasonal variation—the effects of seasonal cycles are easily overlooked.

Perhaps one reason that seasonal effects are underappreciated is because—in contrast to research on other sources of variation (e.g., personality, culture)—research on seasonal effects are scattered across different areas of study that focus on different kinds of outcome variables. For example, research revealing seasonal effects on attentional processes and executive functioning has proceeded largely independently of research revealing seasonal effects on prosociality or sexual behavior. Consequently, these effects, and the specific explanations for them, tend to be conceptually disconnected. This too contrasts with psychological research on other sources of variation (e.g., personality, culture), which are characterized by well-articulated metatheoretical principles of inquiry and conceptual frameworks that integrate findings across diverse research streams. These kinds of frameworks are useful (Muthukrishna & Henrich, 2019). Not only can they help integrate existing findings, but they can also illuminate new research directions and serve as generative foundations for new hypotheses and new empirical discoveries.

If seasonal changes are indeed an important source of human psychological variation, then perhaps “seasonal psychology” merits the kind of scholarly investigation—in terms of both empirical and conceptual inquiry—that has been accorded to personality psychology, cultural psychology, developmental psychology, and other sources of variation. The goal of this article is to encourage a more systematic and comprehensive collective enterprise to reckon with the many ways in which seasons influence human psychology.

## Overview

We begin by briefly highlighting the ubiquity of seasons and the many ways that seasonal variation manifests in people’s lives—including not only changes in meteorological variables but also changes in ecological and sociocultural variables. Next, we summarize some of the many effects that seasons have on a wide variety of psychological phenomena, including mood, aggression, sexual activity, diet and exercise, prosocial behavior, color preferences, and cognitive performance. We then outline a general framework that identifies a set of conceptually distinct causal mechanisms through which seasonal changes—in meteorological, ecological, and sociocultural variables—can influence human affect, cognition, and behavior. We use this framework not only to explain previously documented empirical findings but also to derive novel predictions for additional ways in which seasons may influence psychological phenomena and to identify potential moderating variables (e.g., geographical, cultural, and individual differences) that may influence the magnitude of seasonal effects. In the final substantive section, we draw attention to methodological considerations that follow from this perspective on seasonal variation and offer some practical suggestions for researchers that, we hope, might facilitate additional research on seasonal effects and enhance appreciation for seasons as a fundamental source of variation in human psychology.

## Seasons Are Everywhere and More Than Merely Meteorological

Seasons are most commonly thought of as recurring annual meteorological conditions. This includes changes in features such as sunlight, temperature, and precipitation. In temperate regions, the annual cycle typically includes four seasons (spring, summer, autumn, and winter), each with its own meteorological features; and in the tropics—where there is relatively little annual variation in day length or temperature but considerable variation in precipitation—the year typically comprises a wet season and a dry season. There is additional variability within these four- and two-season conceptualizations. In fact, the Köppen climate classification system—the most widely used climate classification system—outlines 30 distinct seasonal patterns of temperature and precipitation across the globe (Kottek et al., 2006).

Although season cycles are defined by changes in meteorological variables, seasonal variability is characterized by much more than just changes in the weather. As we discuss more fully below, seasonal cycles are also characterized by other kinds of changes that can also affect human experience and human behavior—such as changes in the *ecologies* that people inhabit. Partially as a consequence of changes in meteorological conditions, seasonal cycles produce changes in ecological variables (e.g., prevalence of pathogens, abundance of food and other valued resources) that can have additional, conceptually distinct effects on psychological phenomena. Further, specific seasons are associated with specific kinds of *cultural* traditions, rituals, and large-scale social phenomena (e.g., seasonal holidays and the normative expectations associated with those holidays). Seasonal variation in these cultural variables represents an additional avenue through which seasons can have psychological consequences that are conceptually independent of, and complementary to, effects that are more directly attributable to the weather. Thus, there are multiple reasons to expect seasons to affect a wide variety of human psychological phenomena and multiple routes through which these effects can occur.

## Seasons Are Known to Affect a Wide Range of Psychological Phenomena

In this section, we provide a summary of several lines of empirical evidence documenting the impact of seasons on affective, cognitive, and behavioral phenomena. This overview is by no means exhaustive; it simply illustrates the wide range of ways in which seasonal changes influence psychological phenomena.

### Mood

Black bears can hibernate for up to seven and a half months without eating, drinking, or defecating (Hellgren, 1998). This behavior is viewed as an energy-conserving adaption for when food is scarce in winter. Hibernation has been shown to increase the survival probability of bears and other hibernating animals (Turbill et al., 2011), consistent with this theory. Researchers have noted the similarity between hibernation and SAD. Similar to hibernation, the core symptoms of SAD resemble an energy-conserving strategy (Levitan, 2022): increased eating and sleep, decreased motivation and productivity, and cognitive impairments in learning, memory, and visual-spatial ability (Michalon et al., 1997; O’Brien et al., 1993).

Seasonal effects on mood are also apparent beyond clinical syndromes such as SAD. Researchers have suggested that seasonal variation in mood is common among the general population, and SAD represents an extreme form of this effect (Wehr & Rosenthal, 1989). In fact, one Twitter text analysis of 509 million tweets written by 2.4 million individuals in 84 countries found that decreased day length was associated with less positive affect in tweets (Golder & Macy, 2011), and another analysis of 800 million tweets in the United Kingdom found a peak in sadness in winter (Dzogang et al., 2017). Further, nearly 50% of nondepressed people report experiencing some depressive symptoms in winter (Dam et al., 1998; Kasper et al., 1989), and an analysis of surveys in the United States on subjective happiness from 1946 to 1977 found that happiness was highest in the spring (Smith, 1979).

### Aggression

The summer of 1967 would come to be known as the “long, hot summer.” Riots broke out around the United States, leaving dozens dead and hundreds more injured (Carlsmith & Anderson, 1979). Was the unusually hot summer just the backdrop for the unrest, or did it play a role in what transpired? Psychologists and others were quick to notice the correlation between heat and collective violence. This led to the hypothesis that seasonal increases in ambient temperature cause people to be more irritable, thus increasing the likelihood of aggressive behavior (Anderson, 1989; Miles-Novelo & Anderson, 2022).

This hypothesis has been supported in many studies, including research on riots: As temperatures rose in the summertime, so did the frequency of riots (Gamble & Hess, 2012). Increased ambient temperature has also been linked to many other forms of aggressive behavior, some that are relatively trivial (e.g., horn honking at red lights, acts of ritualized hostility perpetrated in the context of competitive sports; Craig et al., 2016; Krenzer & Splan, 2018; Kenrick & MacFarlane, 1986) and some that are not. For example, crime data from Philadelphia showed that rates of violent crime were 9% higher on the hottest days of the year compared with days at median temperature (Schinasi & Hamra, 2017). More broadly within the northern hemisphere, the summer season from June to August has been associated with higher rates of violent crime (Lauritsen & White, 2014), higher rates of rule infractions in prisons (Haertzen et al., 1993), and higher rates of domestic violence (Sachs & Chu, 2000).

### Sexual activity

Seasons have been linked to variations in the mating behaviors of many animals. Consider the penguin. The emperor penguin (*Aptenodytes forsteri*) and the Galapagos penguin (*Spheniscus mendiculus*) behave similarly in many ways, but they vary drastically in their mating patterns. Emperor penguins live in the Antarctic where day length—and, more pertinently, seasonal supplies of nutrients—varies drastically between summer and winter. Consequently, Emperor penguins breed exclusively in summer when there are plentiful resources to feed newborn chicks. On the other hand, Galapagos penguins live near the equator where this is little seasonal variation in day length and nutritional resources, and they exhibit no particular annual cycle in their mating behavior (Ancel et al., 2013).

Humans appear more similar to Emperor penguins than their cousins in the Galapagos: Humans too exhibit seasonal variation in sexual activity—although the nature of the seasonal cycle is more complex. For instance, in the United States, condom sales, the timing of first intercourse, and Google searches for pornography and prostitution all exhibit a biannual cycle with peaks around Christmas and during the early summer months (Levin et al., 2002; Markey & Markey, 2013; Wellings et al., 1999). These seasonal effects are not small: The biannual cycle accounted for 16% of the variance in pornography searches and 24% of the variance in prostitution searches. Analogous biannual cycles are evident in the timing of diagnoses of sexually transmitted infections (e.g., herpes, syphilis, HIV) and in abortion rates, both of which peak in the months following Christmas and in late summer (Herold et al., 1993; Schroeder et al., 2001).

There are also seasonal cycles in birth rates. In an analysis of 78 years of U.S. monthly natality data, Martinez-Bakker et al. (2014) found that birth rates peaked in the summertime in northern states and peaked in the autumn in southern states. Martinez-Bakker et al. (2014) found that similar seasonal patterns replicated in other countries within the northern hemisphere. These birthrate data suggest that, at least in the northern hemisphere, conception occurs more often in autumn and winter.

### Diet and exercise

Seasonal cycles are also evident in people’s diet and exercise behaviors. Several studies have found that caloric intake reaches its highest peak in autumn and winter (Aparicio-Ugarriza et al., 2017; de Castro, 1991; Ma et al., 2006). Overall diet quality is lower in the winter than any other season, and body weight reaches its peak in winter (Crane et al., 2019; Ma et al., 2006). In addition, physical activity reaches its lowest average in winter and peaks in spring, ostensibly contributing to the lowest maximal oxygen intake and muscle strength in winter (Ma et al., 2006; Shephard & Aoyagi, 2009). These seasonal effects on diet and exercise may contribute to seasonal variation in health-related outcomes such as obesity and rates of cardiovascular events.

Seasonal changes in biological mechanisms have been implicated as potential causes for these effects. For example, Okada (2018) found that satiety after eating increases with outdoor temperatures, suggesting that warm summers contribute to feeling full after a meal. Okada (2018) also found that low humidity increases feelings of hunger, suggesting that the relative aridity that comes with cold weather also contributes to increased eating behavior.

Cultural factors can also play a role in causing seasonal variation in dieting and exercise behavior. For example, an analysis of more than 600,000 tweets on Twitter show that dieting-related tweets peaked in the spring (Griffiths et al., 2022). Further, in a sample of sexual-minority men, Griffiths et al. (2021) found that body dissatisfaction peaked in the summer (especially among men with higher body weight). This seasonal effect on body dissatisfaction was explained by seasonal variation in perceived pressure from media, feelings that one’s body was on public display, and appearance-related social comparisons—all of which also peaked in the summer. Considered together, these results suggest that dieting concerns vary seasonally in anticipation of the body-image concerns associated with summer “swimsuit season.”

### Prosocial behavior

Whereas many seasonal effects in psychological phenomena may be primarily caused by seasonal variation in meteorological conditions or ecological circumstances, others appear to be attributable to season-specific cultural rituals and practices. One example that has been empirically documented in several countries with Christian traditions is a seasonal peak in charitable giving around Christmas—an annual holiday that emphasizes prosocial norms. One analysis found that 29% of annual charitable giving in the United States occurred in the month of December (Network for Good, 2023). Another study analyzed more than 50 million giving decisions over 9 years in Sweden and found a 14% increase in unsolicited charitable donations in December (Ekström, 2018).

This “Christmas effect” on prosociality is not limited just to formal charitable donations; it also appears to manifest on informal forms of everyday prosocial behavior. For example, an analysis of 2 years of within-customer tipping data found that people tip waiters and waitresses more generously during the Christmas holiday season (Greenberg, 2014).

### Color preferences

People’s aesthetic preferences also appear to be influenced by seasonal changes. One illustrative line of research has focused on the colors that people like and dislike. According to the ecological valence theory of human color preferences, such preferences vary as a function of affectively laden cognitive associations with objects in a person’s perceptual environment (Palmer & Schloss, 2010). Those objects (and/or the associations with them) may differ during different seasons, with the implication that affective responses to—and preferences for—colors will also vary seasonally (Palmer & Schloss, 2010; Schloss & Heck, 2017). Using a within-participants longitudinal methodology, Schloss and Heck (2017) found that people living in the northeastern United States responded more favorably to yellow and greenish-yellow colors in the autumn (when those colors may be associated with the splendor of autumn leaves) than in the winter (when they may be associated with less pleasing perceptual stimuli, such as symptoms of sickness), in line with this expectation. Additional studies have also shown that color preferences vary seasonally, in accordance with the predictions of the ecological valence theory of color preferences (e.g., Schloss et al., 2017).

The principles apply not only to objects in the natural ecology but also to colorful artifacts in the cultural environment. For example, in many countries political parties are associated with specific colors; and, because many high-profile political events (e.g., elections) occur cyclically and sometimes take place during specific seasons, this too can lead to seasonal changes in color preferences. Indeed, in the United States preferences for the colors red and blue (symbolically associated with the Republican and Democratic parties, respectively) appear to change on election day in November—and do so in a way that aligns with people’s political-party preferences (Schloss & Palmer, 2014).

### Cognitive performance

Even the most fundamental features of cognitive functioning may be affected by changes in seasons. The cognitive performance of people living with Alzheimer’s disease has been shown to vary seasonally, with more severe symptomatology in winter and early spring (Lim et al., 2018). Additionally, as noted previously, the symptoms of SAD are associated with deficits in working memory, short-term memory, and auditory attention (Merikanto et al., 2012).

Seasonal variation in cognitive performance is not limited to clinical populations. In fact, there is evidence indicating that analogous kinds of seasonal effects may occur more broadly within human populations. For instance, one study—conducted on a sample of healthy young adults in Belgium—found that performance on a measure of sustained attention was highest in the winter and lowest in the summer (Meyer et al., 2016). Additionally, results obtained from the same sample showed that performance on a working-memory task was highest in the autumn and lowest in the spring.

### Summary

As these examples illustrate, changes in seasons influence a wide range of human psychological phenomena (and this variation manifests in ways that are sometimes intriguingly analogous to seasonal patterns of behavior that have been documented in studies of nonhuman animals; see Table 1). The emerging implication is that, perhaps much more than most people are aware, the psychology of *Homo sapiens* is subject to seasonal variation.

**Table 1.** Examples of Seasonal Effects in Human Affect, Cognition, and Behavior (and Some Analogous Examples in Other Animal Species)

Domain of phenomena	Seasonal variation within human populations	Seasonal variation within nonhuman populations
Mood	Winter: decreased mood (Magnusson & Boivin, 2003)	Rhesus macaques—winter: decreased mood (Qin et al., 2015)
Aggression	Summer: increased violence (Anderson, 2001)	Rhesus macaques—autumn (mating season): increased aggression among males (Wilson & Boelkins, 1970)
Sexual activity	December and early summer: increased Internet searches indicating interest in sexual activity (Markey & Markey, 2013)	Emperor penguins—summer (breeding season): increased sexual activity (Ancel et al., 2013)
Diet and exercise	Winter: increased food intake (Ma et al., 2006)	Domesticated cats—autumn and winter: increased food intake (Serisier et al., 2014)
Prosocial behavior	December–January: increased charitable giving (Ekström, 2018)	Prairie voles—winter: increased huddling behavior (Beery et al., 2008)
Color preferences	Autumn: increased preference for yellow (Schloss & Heck, 2017)	Squinting bush brown butterflies—dry season: increased preference for brown (van Bergen & Beldade, 2019)
Cognitive performance	Autumn: increased working-memory performance (Meyer et al., 2016)	African striped mice—winter: increased spatial navigation performance (Maille et al., 2015)

## Mechanisms Through Which Seasons Exert Their Many Effects

**Fig. 1.**
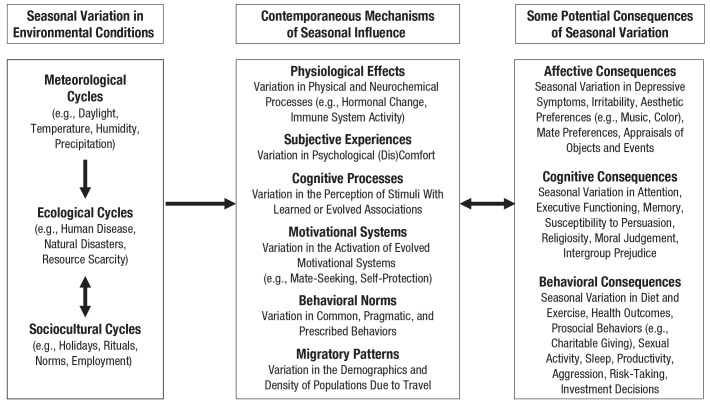
A framework summarizing multiple ways in which seasonal variation manifests in human experience, and multiple causal mechanisms through which these seasonal cycles can influence psychological phenomena.

The preceding section demonstrates that seasons have consequences for a wide array of psychological phenomena—and do so not only as a result of seasonal variation in meteorological variables but also because of seasonal variation in ecological and sociocultural variables. In this section, we elaborate on that latter point by providing an overview of the mechanisms through which seasons can have consequences for affect, cognition, and behavior (these mechanisms are summarized in Fig. 1). As with the preceding summary of empirical findings, this overview is intended to be illustrative rather than exhaustive: It highlights the variety of conceptually distinct mechanisms through which different kinds of seasonal variation—meteorological, ecological, and cultural—can exert effects on different kinds of psychological phenomena. This multimechanism framework may be useful as a means for (a) explaining the extraordinarily wide range of psychological effects that seasons are known to have, (b) reconciling effects that, at first glance, may appear inconsistent, and (c) generating new hypotheses about additional seasonal effects that have not yet been documented in the empirical literature.

### Mechanisms affected by meteorological conditions

For people inhabiting temperate regions of Earth, seasonal changes are most noticeably characterized by changes in hours of daylight, intensity of sunshine, and quantity of heat in the air (all of which are relatively abundant in the summer and scarce in the winter). For people inhabiting the tropics, seasons are experienced differently (e.g., a wet season and a dry season) and are typically defined by differences in rainfall. Exactly how do these meteorological variables produce their cognitive and behavioral consequences? Just as there are multiple meteorological variables and multiple consequences, multiple mediating mechanisms must be considered to fully account for the effects of the former on the latter.

#### Physiological effects (not mediated by cognitive processes)

Meteorological conditions can lead to changes in physiology. In temperate regions, seasonal variation in day length (which typically is associated with variation in sunlight, temperature, and precipitation) is associated with many changes in neurochemical processes within the human body, including processes involving cortisol, μ-opioid reception, serotonin, and testosterone (Demir et al., 2016; Peterson & Harmon-Jones, 2009; Praschak-Rieder et al., 2008; Smals et al., 1976; Sun et al., 2021; Velo et al., 2012). These effects represent multiple neurochemical routes through which seasonal variation in the weather can produce variability in psychological phenomena—and some of these routes can potentially affect a wide range of phenomena.

For example, serotonin binding and serotonin turnover in the brain are influenced by the presence of natural sunlight (e.g., Lambert et al., 2002; Praschak-Rieder et al., 2008). Consequently, in temperate regions, there are effectively higher levels of serotonin in the brain in the summer and lower levels in the winter. Not only does seasonal variation in serotonin help to explain seasonal variation in mood (Praschak-Rieder et al., 2008), it might also help to account for the finding that people are more helpful on sunny days (Cunningham, 1979; Guéguen & Lamy, 2013); and, more generally, seasonal variation in serotonin could have downstream effects on many additional cognitive and behavioral phenomena that are known to be affected by mood—such as memory, risk-taking behavior, and susceptibility to persuasion (e.g., Chou et al., 2007; Eich & Forgas, 2003; Petty & Briñol, 2015).

As with serotonin levels, testosterone levels also tend to be highest in summer months (Smals et al., 1976); and, among men, seasonal increases in testosterone are associated with increased sex drive and sexual behaviors (Demir et al., 2016). Given the wide range of psychological phenomena affected by testosterone, this effect on sexual activity is just one of many potential implications. For example, the experimental administration of testosterone has been shown to increase men’s public displays of charitable giving, increase men’s preferences for high-status consumer products, and increase women’s preference for masculine-looking male faces (Nave et al., 2018; Welling et al., 2007; Wu et al., 2020). These and other psychological phenomena associated with testosterone (e.g. emotion regulation; Kaldewaij et al., 2019) might therefore be expected to vary seasonally—a potentially promising avenue for future research.

#### Effects mediated by subjective experiences

The physiological effects summarized above likely operate outside of conscious awareness, but meteorological variables also have additional consequences that people are aware of and may experience as subjectively pleasant or disagreeable (e.g., when someone is chilled, warm sunshine feels good; when someone is hot, it does not). These subjective experiences can consequently affect the ways in which people appraise objects and events and respond to them.

One illustrative example is provided by research linking hot temperatures to aggressive behaviors. According to Anderson (2001), the process plays out as follows: “Heat-induced discomfort makes people cranky. It increases hostile affect (e.g., feelings of anger), which in turn primes aggressive thoughts, attitudes, preparatory behaviors (e.g., fist clenching), and behavioral scripts (such as ‘retaliation’ scripts). A minor provocation can quickly escalate” (p. 36). The potential consequences of this process are not limited just to aggressive behavior. An analogous explanation accounts for reduced prosocial behavior on uncomfortably hot days (Belkin & Kouchaki, 2017). More generally, because uncomfortably hot temperatures “produce biases in the interpretation of observed social interactions” (Anderson, 2001, p. 36), this subjective experience also has the potential to affect a wide range of judgments about and responses to other people (e.g., the harshness of moral judgments about others’ counternormative actions). The implication for seasonal variation is straightforward: In places where there is seasonal variation in the prevalence of uncomfortably hot weather, there should also be seasonal variation in people’s inclinations toward hostile cognition, harsh judgment, and antisocial behavior.

#### Effects mediated by cognitive associations

Meteorological variables need not elicit specific affective states (e.g., crankiness) to activate specific cognitions that, in turn, facilitate specific behavioral responses. The mere *perception* of a particular meteorological condition might have similar consequences. The underlying principles here are the same as those that inform the ecological valence theory of color preferences (Palmer & Schloss, 2010). People readily learn to associate specific perceptual objects with specific affect-laden concepts (such as the association between yellow and falling leaves; Schloss & Heck, 2017); once such a cognitive association has been acquired, the mere perception of that object makes the associated concept more accessible in working memory, which can then influence attitudes, judgments, and behavioral decisions.

Just as colors can be associated with specific concepts, meteorological variables can also acquire specific cognitive associations. In places where winters are unpleasantly cold, sunshine may acquire positive associations; in places where summers are unpleasantly hot, sunshine may acquire negative associations instead. More universally perhaps, humans may associate daylight with safety and darkness with danger. As a consequence, when people are in the dark (rather than in the light) they respond more fearfully to sudden noises (Grillon et al., 1997) and perceive ethnic out-groups to be less trustworthy (Schaller et al., 2003). Durations of daylight and darkness vary seasonally in temperate regions, with the consequence that people spend more time in the dark during the winter. One implication is that, during winter months, people may be more prone to perceive the potential for danger, with consequences for attitudes and appraisals (e.g., increased risk aversion, greater distrust of strangers).

### Mechanisms affected by seasonal variation in local ecologies

Just as the weather changes with the season so too do the ecologies inhabited by humans. Many plants produce foliage seasonally (e.g., in tropical climates, some species of trees are leafless during the dry season and fully foliated during the rainy season), creating seasonal variation in the lushness of the local landscape. Additionally, in many parts of the world there is seasonal variation in the prevalence of infectious diseases such as influenza, cholera, and malaria (Martinez, 2018). These and other seasonal changes in local ecologies have the potential to influence human affect, cognition, and behavior—and to do so in ways that are conceptually distinct from, and complementary to, the effects of meteorological variables.

#### Physiological effects (not mediated by cognitive processes)

Beyond the physiological effects of the weather (e.g., light, temperature), seasonal variation in the local ecology can also influence human physiology. One illustrative—and speculative—example of the mediating role of ecology follows from a joint consideration of seasonal variation in the incidence of infectious diseases (Martinez, 2018) and research on cytokine-induced sickness behavior (Dantzer & Kelley, 2007). Seasonal variation in the prevalence of infectious diseases implies seasonal variation in the percentage of people within a population who contract infections—and thus seasonal variation in the extent to which populations exhibit sickness-related behaviors. These effects are not limited just to physical symptoms of illnesses; they include psychological effects as well. When the immune system detects the presence of an infection, it typically mounts an inflammatory response that is mediated by the release of proinflammatory cytokines. These cytokines do more than merely promote inflammation. They also exert diverse effects on the peripheral and central nervous systems (Hopkins & Rothwell, 1995; Rothwell & Hopkins, 1995), leading to a diverse range of cognitive and behavioral outcomes—including reduced motor activity and social withdrawal—that have, collectively, been called “sickness behavior” (Dantzer & Kelley, 2007; Hart & Hart, 2019). This process may contribute to the pathogenesis of depression—as indicated, for instance, by empirical evidence linking inflammation to negative mood states and major depressive disorder (Miller & Raison, 2016).

In addition to these clinical implications, inflammation “may play an important modulatory role in shaping emotions, motivation, cognition, and behavior” and, as a consequence, “may be an important mediator of many psychological and behavioral outcomes that are of interest to social and personality psychologists” (Gassen & Hill, 2019, p. 1). For example, the experimental induction of a benign endotoxin—which stimulates an inflammatory response without introducing a harmful infection—caused people to report greater desire to spend time with supportive friends and family members (Inagaki et al., 2015). These and other findings (for a review, see Gassen & Hill, 2019) indicate that immunological responses to infection may have subtle and nuanced consequences for human cognition and behavior. The further implication is that immunological mechanisms may provide one specific route through which one specific kind of seasonal change in the local ecology—variation in the prevalence of infectious diseases (Martinez, 2018)—might also have subtle and nuanced consequences for human cognition and behavior.

#### Effects mediated by the activation of specific motivational systems

Another route through which seasonal changes in local ecologies can have consequences is through the activation of specific motives and goals. Humans are equipped with motivational systems that evolved to regulate behavioral responses to one’s environment (Schaller et al., 2017; Tooby et al., 2008). These motivational systems are attuned to perceptual cues connoting the presence of specific threats to be avoided or specific opportunities to be seized. These appraisals, in turn, stimulate functionally specific affective, cognitive, and behavioral responses. For instance, the sound of a sudden roar connotes the presence of a predator and thus activates motivational mechanisms that regulate self-protective responses (Neuberg et al., 2011), whereas the sight of neonatal facial features connotes the presence of an infant and activates motivational mechanisms that regulate parental caregiving responses (Schaller, 2018). To the extent that there is seasonal variation in these and other motive-activating cues within the local ecology, then there may be seasonal variation in the activation of specific motivational systems—and thus also seasonal variation in their characteristic emotional, cognitive, and behavioral responses.

To illustrate, consider again the fact of seasonal variation in the incidence of infectious diseases (Martinez, 2018) and its implications for the activation of the motivational system that regulates proactive behavioral defenses against infection (the “behavioral immune system”; Schaller & Park, 2011). This motivational system is more readily activated under circumstances in which individuals perceive themselves to be more vulnerable to infection; and once activated, it has consequences for a wide range of cognitive and behavioral phenomena (Ackerman et al., 2018; Murray & Schaller, 2016; Schaller et al., 2022). For example, when people feel more vulnerable to infection, they consequently are more distrustful of other people (Aarøe et al., 2016), reluctant to pursue dating relationships (Sawada et al., 2018), troubled by crowds (Wang & Ackerman, 2019), wary of used consumer products (Huang et al., 2017), xenophobic (Faulkner et al., 2004), likely to conform to majority opinion (Murray & Schaller, 2012), likely to judge moral transgressions harshly (Murray et al., 2019), and supportive of conservative sociopolitical policies (Aarøe et al., 2020). Assuming that people generally perceive themselves to be more vulnerable to infection when they actually are more vulnerable to infection, then analogous seasonal effects might plausibly occur as a consequence of seasonal variation in the incidence of infectious diseases in the local ecology.

#### Effects mediated by cognitive associations

Seasonal variation might also be produced by a process in which specific ecological features are cognitively associated with specific concepts and consequently facilitate cognitive access to those concepts. In the preceding section we summarized how this process can lead to seasonal variation in color preferences (the ecological valence theory of color preferences; Palmer & Schloss, 2010), but the potential consequences are not limited just to color preferences. There are implications for seasonal variation in many psychological phenomena.

To illustrate, consider the outcomes of perceived resource scarcity. Experiments that manipulate perceptions of abundance and/or scarcity have shown that these perceptions can affect a wide range of phenomena, including object attachment (Goldsmith et al., 2021), categorization of racially ambiguous faces (Rodeheffer et al., 2012), inclinations toward impulsiveness and risky decision-making (Griskevicius et al., 2013), and prosocial behavior (Roux et al., 2015). In real life, the concepts of resource abundance and resource scarcity may be associated with seasonally variable ecological circumstances. In temperate regions, for instance, the lush vegetation of summer may tacitly connote abundance, whereas bleak wintertime landscapes are more likely to be associated with scarcity. Consequently, when wintery—rather than lush—landscapes are perceived, the concept of scarcity may be more readily accessible in working memory, with implications for seasonal variability in the many cognitive or behavioral phenomenon that are affected by perceptions of scarcity (e.g., object attachment, face categorization, risky decision-making, prosocial behavior).

#### Effects mediated by changes in the social ecology

The preceding paragraphs focused on seasonal variation in the *natural* ecology—the pathogens, plants, and other nonhuman organisms that characterize a person’s local environment. A person’s local ecology is defined also by the other people who occupy their local environment. In other words, in addition to the natural ecology, the *social* ecology matters as well (Oishi & Graham, 2010; Sng et al., 2018; Stokols, 1992; Uskul & Oishi, 2020). Just as a person’s natural ecology can vary seasonally, a person’s social ecology may also vary seasonally, simply as a consequence of season-specific patterns of human travel and migration.

Consider, for example, the “Spring Break” phenomenon during which many beach communities in the southern United States and Mexico are temporarily inhabited by throngs of young adults in skimpy swimwear. This seasonal change in local social ecology may create a salient social-comparison context that temporarily arouses body-image anxieties, especially among young women (Griffiths et al., 2021), which may produce season-specific changes in the problematic sequelae associated with these anxieties (e.g., restrained eating, disrupted academic performance; Fredrickson et al., 1998; Quinn et al., 2006).

Some social-ecological variables—such as population density—may vary seasonally in places that are popular destinations for seasonal travel. For instance, the Bahamas are most densely populated during winter, Provençal villages are most densely populated during summer, and Mecca is most densely populated during the H.ajj. High population density has psychological consequences, some that have been well known for decades (e.g., the experience of crowding is subjective unpleasant and stressful; Baum & Valins, 1979) and some that have only recently been illuminated. For instance, experiments show that the mere perception of greater population density can lead people to become more future-oriented, to prefer fewer lifetime relationship partners, and to prefer having fewer children (Sng et al., 2017). Some seasonal travel choices may be particularly popular among people with specific demographic characteristics. Consequently, the age profile of a local population may vary seasonally (e.g., the wintertime influx of retirees to many parts of Arizona), and even the sex ratio may vary seasonally in some places. These changes may also have subtle consequences for the thoughts and actions of the people who inhabit these ecologies (Sng et al., 2018).

### Mechanisms affected by seasonal variation in cultural events and practices

Festivals and holidays (e.g., Tsagaan Sar, Diwali, Christmas) occur during specific times of the year. This creates seasonal variability in ritualized behaviors (e.g., gift giving) and in the perceptual stimuli to which people are incidentally exposed (e.g., Santa Claus). Schools are in session during specific chunks of the year and not others, contributing to seasonal variability in the amount of time that parents and children spend together. The frequencies of many other cultural practices also vary seasonally (e.g., weddings, pool parties, hunting trips). These cultural events and popular practices can have additional effects on human cognition and behavior that are distinct from, and conceptually complementary to, the effects resulting from seasonal changes in the weather or local ecology.

#### Effects mediated by the activation of specific motivational systems

Just as motivational systems can be activated by cues in the local ecology (as discussed above), they can also be activated by specific kinds of cues associated with cultural events and practices; and if there is seasonal variation in these social cues, it may lead to seasonal variation in the activation of specific motivational systems and in their characteristic emotional, cognitive, and behavioral responses.

To illustrate, consider school holidays. Compared with those times of year during which schools are in session, parents of school-aged children are likely to spend more time actively inhabiting their role as parents during school holidays. This may lead to increased activation of the motivational system that governs parental caregiving behavior and kin care generally (Schaller, 2018). As a consequence, parents might plausibly show seasonal variation across a wide range of affective, cognitive, and behavioral phenomena that have been shown to be affected by this motivational system—including not only caregiving behavior but also aggression, risk aversion, intergroup prejudice, mate preferences, moral judgment, and sociopolitical attitudes (Buckels et al., 2015; Eibach et al., 2009; Gilead & Liberman, 2014; Hahn-Holbrook et al., 2011; Kerry & Murray, 2020).

Other seasonal effects might possibly be produced by seasonal changes in clothing norms and their implications for the activation of motivational mechanisms underlying mating behavior. In temperate climates, people are more likely to wear scanty attire in the summer. Motivational mechanisms underlying mating behavior respond to visual cues of this sort, with the implication that mating motives may be more readily activated in the summer—perhaps especially among people who are most sensitive to such cues (e.g., adolescents and young adults). The activation of mating motives not only has implications for overt acts of mating behavior but can also have a wide range of additional cognitive and behavioral consequences. For instance, the activation of mating motives in young men has been shown to increase their tendencies toward conspicuous consumption and blatant benevolence (Griskevicius et al., 2007); creativity, nonconformity, and risk-taking behavior (e.g., Griskevicius, Cialdini, & Kenrick, 2006; Griskevicius, Goldstein, et al., 2006; Ronay & von Hippel, 2010; Wilson & Daly, 1985); and biased judgments about other people (e.g., Maner et al., 2005). For people who are already in a mating relationship, the popular practice of shedding clothes in the summertime also increases exposure to scantily clad bodies of people that their own mates might find sexually attractive. This can activate motivational mechanisms that regulate mate-retention behavior—which may manifest in jealousy and mate-guarding behavior (Buss & Haselton, 2005), as well as in increased attention to, and distrust of, individuals who might be especially attractive to one’s mate (Krems et al., 2016; Maner et al., 2007). Thus, there also might be seasonal variation in these phenomena.

#### Effects mediated by cognitive associations

We have already discussed a process through which seasonally variable features of the weather and local ecology can be cognitively associated with specific concepts and thus facilitate cognitive access to those concepts, with downstream consequences for judgment and behavior. This process applies to seasonal variation in cultural practices too. For instance, in North America, the Thanksgiving and Christmas holidays have become associated with the concepts of benevolence and generosity (e.g., giving thanks, giving gifts). Consequently, the mere awareness of the holiday has the potential to make those concepts—along with other thoughts and beliefs associated with them—more cognitively accessible. This may have consequences not only for overt acts of benevolence (e.g., giving money to charitable causes; Ekström, 2018) but also for less obvious manifestations of a generous mindset (e.g., more forgiving responses to others’ misdeeds). The implication for seasonal variation is that regardless of meteorological conditions, whenever holidays of this sort are psychologically salient (e.g., because of holiday themed content in popular media) people may exhibit more giving—and forgiving—thoughts and behaviors.

#### Effects mediated by seasonal changes in normative behavioral choices

For pragmatic reasons, people often make different behavioral choices during different seasons. Those popular choices represent seasonal changes in normative behaviors that may have additional (often unintended) consequences for cognition and behavior for reasons that are conceptually distinct from the processes outlined in the preceding paragraphs.

For instance, seasonal variation in the harshness of meteorological conditions creates seasonal variation in the amount of time that people spend outdoors and in nature. Exposure to nature has psychological consequences—including benefits for attention and memory and for health and well-being (e.g., Berman et al., 2008, 2012; Hartig et al., 2014). The implication is that, in places where people spend disproportionately more time in nature during some seasons rather than others, one might also expect there to be some seasonal variation in attention, memory, health, and well-being. Nature may even provide a kind of spiritual resource, decreasing the subjective need to seek spiritual comfort through other means. In fact, some work has found lower adherence to traditional religions in places where people have greater access to nature (Ferguson & Tamburello, 2015). If there is merit to this analysis, one implication might be that in places characterized by seasonal variation in access to nature, they may also be seasonal variation in traditional forms of religious activity.

These are just a few speculative examples that illustrate a broader point: Large numbers of people make similar season-specific behavioral choices; and when they do, it may produce additional seasonally variable effects on human affect, cognition, and behavior.

## Moderators of Seasonal Effects

As summarized in the preceding sections, seasonal cycles manifest in a multitude of ways that can have effects on a wide range of psychological phenomena. But the *magnitude* of these seasonal effects likely varies, depending on other variables that differ across contexts or across individuals. In this section, we provide a brief overview of some variables that might be expected to moderate the impact that seasons have on affect, cognition, and behavior.

### Geographical variables

Any seasonal effect attributable to variation in meteorological conditions is likely to be greater in places that are subject to more extreme variations in those conditions. Residents of both Kugluktuk, Nunavut (in northern Canada) and Paducah, Kentucky (in the continental United States) experience seasonal variation in sunlight and ambient temperature; however, because of a substantial difference in latitude (67°N and 39°N, respectively) that variability is more extreme in Kugluktuk than Paducah. To the extent that these meteorological variables affect psychological phenomena, the magnitude of those effects would be expected to be bigger in Kugluktuk—and, more generally, in locations characterized by a higher latitude. This kind of moderating effect has been observed in the experience of SAD, which is more prevalent at higher latitudes (Kegel et al., 2009; Mersch et al., 1999).

Higher latitudes are characterized not just by more extreme seasonal variation in meteorological variables but also by more extreme variation in their ecological consequences (e.g., greater scarcity of plant life in the wintertime, which may contribute to the subjective appraisal of a harsher environment). It is because of these kinds of ecological covariates, and not just meteorological covariates, that latitude predicts cross-national differences in human behavior (e.g., Van de Vliert, 2020; Van de Vliert et al., 2023; Van de Vliert & Van Lange, 2019; Van Lange et al., 2017). If indeed seasonal variation in local ecologies accounts for some seasonal effects on psychological outcomes, and if indeed the extremity of that variation differs at different latitudes, then the sizes of those effects would also be expected to differ at different latitudes.

The magnitude of seasonal variation is also affected by other geographical variables, such as proximity to ocean waters. Moscow and Copenhagen are at the same latitude (55°N); however, compared with residents of coastal Copenhagen, residents of Moscow experience hotter summers and colder, snowier winters—which implies greater variation in the perceived harshness of the landscape. To the extent that those forms of seasonal variation have psychological consequences, those effects may be bigger among Moscovites. The same principle applies to tropical seasons. Quito and Guayaquil—the two largest cities in Ecuador—are both located close to the equator but are geographically different in other ways (Guayaquil is coastal; Quito is in the highlands). As a consequence, Guayaquil has both a drier dry season and wetter wet season. Any psychological consequences of the dry/wet seasonal cycle are likely to be greater in Guayaquil.

The broader point is this: Geography matters. In addition to the many main effects that geographical variables have on psychological phenomena (e.g., Götz et al., 2020; Rentfrow, 2020), these variables also have implications for the magnitude of seasonal variation in meteorological and ecological conditions. As a consequence, geographical variables may moderate the magnitude of seasonal effects on affect, cognition, and behavior.

### Demographic variables that affect a person’s exposure to seasonal variation

Seasonal variation in meteorological and ecological variables can affect people only to the extent that people are actually exposed to that variation. That exposure differs depending on a person’s demographic circumstances, such as whether they live in a rural or urban environment, or whether they are rich or poor.

Compared with urban populations, people in rural areas are more likely to work in industries (e.g., agriculture) that are directly affected by the meteorological and ecological manifestations of the seasons, and they spend more time outdoors (Matz et al., 2015). As a consequence, exposure to seasonal variation is likely to be greater in rural populations. Indeed, there is evidence that inhabitants of rural areas are more susceptible to certain kinds of seasonal effects. For example, a study in Finland found more severe behavioral symptoms of SAD in rural areas (Sandman et al., 2016), and a study in Italy found that seasonal variation in suicide rates was also more pronounced in rural populations (Micciolo et al., 1991).

Regardless of whether a person inhabits a rural or an urban environment, that person is more likely to be exposed to seasonal variation (e.g., hot summers and cold winters) if they lack resources that provide buffers against that variation (e.g., air-conditioned cars, well-insulated homes). Access to those resources is a function of wealth. The implication is that some seasonal effects on psychological phenomena—perhaps especially effects resulting directly from variation in meteorological variables—may be larger among people who are impoverished.

### Trait-like individual differences

People differ in their sensitivities to different kinds of seasonal variation, and these trait-like differences are therefore likely to moderate the effects of seasons on thoughts, feelings, and actions. Research on SAD provides an illustrative example, showing that people differ in their vulnerability to seasonal depression and its sequelae (Levitan, 2022). Likewise, Bronson (2004) made the case that individuals differ in sensitivity to day length, with implications for individual differences in the magnitude of seasonal effects on sex hormones and reproductive outcomes. Individual differences in sensitivity to heat, humidity, and other meteorological variables might be expected to moderate seasonal effects on aggression and other outcomes mediated by physical discomfort. People also differ in their sensitivity to variation in social norms and expectations, and in their eagerness to comply with those expectations (e.g., Gangestad & Snyder, 2000). These individual differences may moderate seasonal effects that result from the specific norms and expectations—and associated cognitions—that accompany seasonal cultural events (such as Ramadan or Diwali).

Other kinds of individual differences may also matter. Some of the hypotheses identified earlier in this article are predicated on seasonal variation in exposure to specific kinds of stimuli that trigger specific kinds of cognitive associations (e.g., the iconography of Christmas may activate cognitions associated with gift giving, with implications for benevolence more generally). The strength of these associations can vary across individuals depending on their previous experiences and learning histories, implying individual differences in the size of the hypothesized effects. Other hypotheses are predicated on seasonal variation in stimuli that activate specific motivational systems (e.g., darkness activates a self-protective motive; the presence of sick people activates a disease-avoidance motive). Individuals differ in the extent to which these motivational systems are readily activated (Neel et al., 2016). For example, some people feel more vulnerable to disease than others (Duncan et al., 2009); thus, if seasonal variation in sickness leads to seasonal variation in disease-avoidant cognitions and behavior, these effects may be more evident among individuals who feel more vulnerable to infection. More generally, individual differences in the strength of specific cognitive associations and specific motives may moderate specific effects resulting from seasonal variation in specific kinds of perceptual stimuli.

### Cultural differences

Just as the psychological effects of seasons may be moderated by individual differences, so too may they be moderated by analogous cultural differences. For example, cultures differ in their level of industrialization and infrastructure (e.g., climate-controlled office buildings, covered bus shelters) that reduces individuals’ exposure to the meteorological and ecological manifestations of seasons. The implication is that some seasonal effects may be more pronounced in less industrialized cultures. Population-level economic variables—which can interact with climatological variables to predict behavioral outcomes (Van de Vliert, 2013; Van de Vliert et al., 2023; Van de Vliert & Murray, 2018)—might also moderate some effects of seasonal variation on psychological phenomena.

One might also expect cultural differences in the magnitude of effects that result from seasonal variation in the activation of specific motivational systems, simply because cultures differ in the motivational profiles of their resident populations (Pick, Ko, Kenrick, et al., 2022; Pick, Ko, Wormley, et al., 2022). Cultures also differ in the kinds of perceptual stimuli and normative expectations that accompany seasonal cultural events. For example, although Christmas is celebrated in many countries, the popular iconographies of Christmas (e.g., visual depictions of St. Nicholas, Father Christmas, and/or Santa Claus) vary across those countries, as do Christmas rituals and expectations. If indeed these kinds of cultural stimuli and normative expectations account for some seasonal effects on psychological phenomena, then these effects too may be stronger in some cultures than others.

## Toward a Systematic Science of “Seasonal Psychology”

In the preceding sections we have attempted to highlight the many effects that seasonal cycles have on psychological phenomena and the variety of underlying causal mechanisms through which these effects may occur. One implication (which we have illustrated by offering speculative hypotheses that still remain to be tested) is that existing evidence of seasonal effects may represent just a preliminary peek at the full extent to which seasonal cycles influence human psychology. If so, then there are also *practical* implications for scientists who study human psychology. For example, because unaccounted-for variability (i.e., “noise”) in empirical data depresses statistical power, psychological scientists might be wise to carefully consider inferential costs that might accrue from failures to anticipate, and analytically account for, seasonal variation in the data that they collect. And, if indeed there are (as we suspect) many psychological effects of seasons that still remain to be discovered, then it might also be useful for more psychological scientists to engage in more systemic efforts to discover them.

It is with these implications in mind that, in this section, we briefly summarize a set of practical suggestions. Some suggestions are directed toward researchers who, like us, are intrigued by the potential for a systematic science of “seasonal psychology,” are inspired to initiate new studies that might reveal new effects of seasons on psychological variables, and who might benefit from guidance about resources and tools that might aid those endeavors. Additionally, we offer several suggestions for data collection and reporting practices that all researchers (regardless of personal interest in seasonal phenomena) can engage in to help ensure that the collective database of the psychological sciences is more systematically attentive to the effects of seasons on human psychology.

### Useful resources, tools, and techniques

To document seasonal effects, it is necessary to conduct analyses on data collected across multiple seasons. Further, to test the replicability and robustness of those effects, it may be necessary to conduct analyses on data collected across multiple years. Until recently, these inferential requirements posed a substantial deterrent to the systematic study of seasonal psychology. But times have changed. Advances in online data-collection methods now make it relatively easy to collect psychological data from large samples over long periods of time; and there are many readily available sources of “big data” that can also be analyzed for seasonal trends (Adjerid & Kelley, 2018; Rafaeli et al., 2019). For instance, researchers now have the opportunity to access results of many large-scale data-collection projects (e.g., Project Implicit, Gosling Potter Internet Personality Project, YourMorals.org, Moral Machine), for which data have been collected from millions of participants over months, years, and sometimes even decades. Researchers can also easily access social-media and search-engine data (e.g., Twitter, Reddit, Google) that can be mined to test for temporal trends, and recent studies have productively seized on this opportunity to document seasonal cycles in psychological phenomena (e.g., Griffiths et al., 2022; Hamamura & Chan, 2020).

There are unique inferential challenges associated with analyses of temporal trends in data that may seem daunting to researchers who are new to the study of seasonal variation. But these challenges need not pose an obstacle; statistical tools are available to make rigorous analyses of seasonal cycles relatively easy to learn and execute (for a useful and not-too-daunting introduction to some of these inferential challenges and analytic means of addressing them, see Jebb et al., 2015). Multiple powerful methodologies can be used to test for seasonal patterns in psychological data (e.g., harmonic regression, ARIMA modeling, prophet modeling), and readily accessible R coding packages (e.g., *rHarmonics*, *forecast*, *prophet*) are available to researchers who wish to implement them.

Researchers who study seasonal variation often face decisions about appropriate units of analysis. For example, should calendar day be treated as the unit of analysis, or should data be aggregated at the level of week, or month, or some other temporal unit? These analytic decisions can have nontrivial inferential implications (e.g., aggregating data into larger temporal units typically improves measurement reliability but reduces degrees of freedom—both of which have implications for statistical power). In many ways, these decisions about *temporal* units are analogous to the decisions about *geographical* units (e.g., postal code, state, country) that must be made by researchers who study regional or cultural variation in psychological phenomena. Recent advances in geographical psychology address the importance of thoughtfully selecting geographical units of analysis that are appropriate to particular hypotheses or research questions (Ebert et al., 2022), and these analytic considerations may also help guide the analytic decisions made by seasonal researchers. For this reason—and because seasonal effects may be moderated by geographical variables (as discussed above)—researchers studying seasonal variation may obtain useful methodological guidance not only from the existing research on seasonal effects but also from research in cultural and geographical psychology.

Even if a seasonal effect is observed in some psychological phenomenon, it can be challenging to infer the mechanism(s) through which that effect occurs. We have outlined many possible sources of seasonal variation, involving meteorological, ecological, and cultural variables that often covary and are difficult to disentangle. To help with this thorny inferential task it can be useful to systematically assess, and compare, effects within multiple geographical locations. For example, if a seasonal effect observed in the northern hemisphere is caused by cyclical changes in meteorological variables (e.g., day length), the timing of that effect—measured in terms of calendar dates—would be expected to reverse in the southern hemisphere. Useful clues can also be provided by careful empirical attention to other geographical variables of the sort discussed above—such as latitude, which has implications for the extremity of seasonal changes in specific meteorological and ecological variables and thus for the sizes of effects of those variables. In contrast, if an effect results from cyclical changes in specific cultural practices (e.g., holidays), one would not expect it to be moderated by latitude but instead by cultural differences in the presence or absence—or the specific timing—of those practices. It can also be useful to systematically assess, and compare, seasonal effects across multiple years. For example, if a seasonal effect is caused by cyclical changes in a particular ecological variable (e.g., disease prevalence), relevant evidence might include a comparison of effect sizes across years with naturally varying levels of that variable (e.g., years with especially low vs. high levels of seasonal influenza). Additionally, because naturalistic studies of seasonal effects are inevitably correlational, it can be inferentially useful to complement them with laboratory experiments. Even though annual seasonal cycles cannot be realistically manipulated within an experiment, it is possible to experimentally manipulate some specific variables that vary seasonally (e.g., ambient temperature, temporary perceptual exposure to lush vs. harsh landscapes), and the results of these experiments may bear indirectly on specific underlying mechanisms.

### Data collection and reporting practices

We are struck by historical parallels between the study of seasonal variation and the study of cultural variation. Just as seasonal effects are often overlooked as a fundamental source of variation in psychology, there was a time when cultural differences were similarly underappreciated and overlooked. The belated emergence of a truly systematic science of cultural psychology required that large numbers of researchers were sensitive to the possibility of cultural differences and designed empirical studies to obtain data from multiple cultures. The collection of data from multiple cultures is now recognized as a methodological imperative within psychology and the behavioral sciences more broadly (Apicella et al., 2020; Henrich et al., 2010). Analogously, the emergence of a truly systematic science of seasonal psychology will require that large numbers of researchers be sensitive to the possibility of seasonal effects and design empirical studies to obtain data across multiple seasons. Indeed, this too might be viewed as an imperative. At the very least, when analyzing data that were collected over spans of months or more, researchers can test for seasonal effects. Even better, researchers can proactively design studies so that data are collected across multiple seasons, test for seasonal differences (even if only in an exploratory way), and make the results (and the data) publicly available. By deliberately collecting data that might potentially document the presence or absence of seasonal effects, researchers—even those whose primary interests lie elsewhere—contribute to the systematic study of seasonal variation in psychology.

Finally, every psychological scientist who does any empirical study can contribute to the systematic study of seasonal variation by doing one very simple thing: When reporting on the study, report *when* the data were collected. It is already common practice to report *where* data were collected, and there are good reasons to do so (e.g., this information has implications for the generalizability of findings, interpretation of replication attempts, and can be used to test for regional and/or cultural differences in subsequent meta-analyses). For analogous reasons, it would also be good practice to report when those data were collected. Doing so provides information that may have implications for the generalizability of findings (perhaps especially when the phenomenon of interest is already known to be seasonally variable), aids in the interpretation of subsequent replication studies (regardless of the outcomes of those studies), and allows for subsequent meta-analyses to test for seasonal effects. These and other benefits are essential to a systematic science of seasonal psychology. Additionally, given the potential for specific geographic and demographic variables (e.g., latitude, population density) to moderate effects of seasonal cycles, it would also be good practice to be as precise as possible (e.g., Hanoi, Hokkaido, New Hampshire) rather than vague (Vietnam, Japan, the United States) when reporting where data were gathered. This too will help support rigorous and systematic inquiry.

## Conclusion

Almost a century ago, in an article on the seasonal factor in human culture, Thomson (1939, p. 209) wrote that seasons had such a powerful effect on the behavior of nomadic tribespeople that, when observed during different seasons, these people appeared to be different people entirely:

It will be apparent that an onlooker, seeing these people at different seasons of the year, would find them engaged in occupations so diverse, and with weapons and utensils differing so much in character, that if he were unaware of the seasonal influence on food supply, and consequently upon occupation, he would be led to conclude that they were different groups.

If anything, Thomson’s observations probably underestimate the impact of seasons on *Homo sapiens*. Seasons exert profound influences on contemporary populations of people all over the world, and seasonal effects occur across an extraordinarily wide range of psychological phenomena. In short, seasons appear to be a fundamental source of variability in how people think, feel, and behave. For many years, variability because of *place* (i.e., geography, culture, ecological conditions) was largely overlooked in the theorizing and methodology of mainstream psychology, and the psychological literature is richer now that it is no longer the case. We are of the view that the impact of *time* (Varnum & Grossmann, 2017)—and *time of year* in particular—has similarly been underappreciated and that our understanding of human psychology will be improved if more widespread efforts are undertaken to undo this oversight. To the extent that seasons create variability in psychological phenomena, then it behooves researchers to be attentive to that variability and, ideally, to measure it, account for it, and understand it. Rather than remaining an overlooked source of noise in the database of the psychological sciences, seasonal variation can instead be systematically documented as a key source of signal—a fundamental feature of human psychology.

## Transparency

*Action Editor:* Tim Pleskac

*Editor:* Interim Editorial Panel

*Author Contributions*

I. Hohm and A. S. Wormley contributed equally to this manuscript. M. E. W. Varnum, M. Schaller, I. Hohm, and A. S. Wormley conceptualized this manuscript. I. Hohm, A. S. Wormley, M. Schaller, and M. E. W. Varnum wrote the initial draft and contributed to manuscript revisions. M. E. W. Varnum and M. Schaller supervised the project. All of the authors approved the final manuscript for submission.
